# A novel cardiac output response to stress test developed to improve diagnosis and monitoring of heart failure in primary care

**DOI:** 10.1002/ehf2.12302

**Published:** 2018-06-26

**Authors:** Sarah J. Charman, Nduka C. Okwose, Renae J. Stefanetti, Kristian Bailey, Jane Skinner, Arsen Ristic, Petar M. Seferovic, Mike Scott, Stephen Turley, Ahmet Fuat, Jonathan Mant, Richard F.D. Hobbs, Guy A. MacGowan, Djordje G. Jakovljevic

**Affiliations:** ^1^ Cardiovascular Research Centre, Institutes of Cellular and Genetic Medicine, Faculty of Medical Sciences Newcastle University Newcastle upon Tyne UK; ^2^ Wellcome Trust Centre for Mitochondrial Research, Institute of Neuroscience, Medical School Newcastle University Newcastle upon Tyne UK; ^3^ Royal Victoria Infirmary, Newcastle upon Tyne Hospitals NHS Foundation Trust Newcastle upon Tyne UK; ^4^ Cardiology Department, Clinical Centre Serbia, School of Medicine University of Belgrade Belgrade Serbia; ^5^ Newburn Surgery Newcastle upon Tyne UK; ^6^ Roseworth Surgery Newcastle upon Tyne UK; ^7^ Darlington Memorial Hospital, County Durham and Darlington NHS Foundation Trust and School of Medicine, Pharmacy and Health Durham University Durham UK; ^8^ Primary Care Unit, Department of Public Health and Primary Care University of Cambridge Cambridge UK; ^9^ Nuffield Department of Primary Health Care Sciences University of Oxford Oxford UK; ^10^ RCUK Centre for Ageing and Vitality Newcastle University Newcastle upon Tyne UK

**Keywords:** Heart failure, Diagnosis, Primary care, General practice, Test, Cardiac output, Reproducibility

## Abstract

**Aims:**

Primary care physicians lack access to an objective cardiac function test. This study for the first time describes a novel cardiac output response to stress (CORS) test developed to improve diagnosis and monitoring of heart failure in primary care and investigates its reproducibility.

**Methods and results:**

Prospective observational study recruited 32 consecutive primary care patients (age, 63 ± 9 years; female, *n* = 18). Cardiac output was measured continuously using the bioreactance method in supine and standing positions and during two 3 min stages of a step‐exercise protocol (10 and 15 steps per minute) using a 15 cm height bench. The CORS test was performed on two occasions, i.e. Test 1 and Test 2. There was no significant difference between repeated measures of cardiac output and stroke volume at supine standing and Stage 1 and Stage 2 step exercises (all *P* > 0.3). There was a significant positive relationship between Test 1 and Test 2 cardiac outputs (*r* = 0.92, *P* = 0.01 with coefficient of variation of 7.1%). The mean difference in cardiac output (with upper and lower limits of agreement) between Test 1 and Test 2 was 0.1 (−1.9 to 2.1) L/min, combining supine, standing, and step‐exercise data.

**Conclusions:**

The CORS, as a novel test for objective evaluation of cardiac function, demonstrates acceptable reproducibility and can potentially be implemented in primary care.

## Introduction

Widely considered the new epidemic of the 21st century, heart failure is a complex clinical syndrome associated with structural and functional cardiac abnormalities, which lead to reduced cardiac output at rest and/or stress.^1^ Heart failure increases with age and in the presence of comorbidities^2^, ^3^ and is a leading cause of hospitalization in people over 65 years of age.^1^, ^2^ The prevalence of cardiac dysfunction and associated heart failure is >10% in people ≥70 years of age and rises to >20% in those with multi‐morbidities including diabetes, coronary artery disease, metabolic syndrome, hypertension, and rheumatoid arthritis.^1^ Heart failure is associated with poor prognosis, poor quality of life for patients, and high healthcare costs.^1^, ^2^

Early diagnosis of heart failure is crucial because prompt initiation of treatment significantly improves morbidity, quality of life, and mortality, while reducing hospitalization and healthcare costs.^1^, ^4^ In the absence of acute onset of heart failure, patients commonly present to primary care with signs and symptoms such as ankle swelling, breathlessness, and fatigue.^2^ These are non‐specific and do not, therefore, help discriminate between heart failure and other problems associated with age, obesity, and lung disease.^3^

General practitioners (GPs) have a key role in the early identification of heart failure.^3^, ^5^ However, currently available diagnostic tools, such as electrocardiography, are insufficiently sensitive to detect heart failure.^6^ Although the serum natriuretic peptides test is more accurate as a ‘rule out’ test to exclude heart failure, it can lead to a significant number of false positive results due to its overall reduced specificity.^7^, ^8^, ^9^ An increased serum natriuretic peptide level is not only found in patients with heart failure but also in those living with chronic pulmonary disease, left ventricular systolic dysfunction, renal failure, left ventricular hypertrophy, coronary artery disease, elderly, and obese. It is thus not surprising that current primary care clinical practice for heart failure results in inaccurate and expensive referrals to secondary care, which creates additional worry for the patient. It is suggested that the majority of patients (>65%) suspected of having heart failure by their GPs (based on symptoms, signs, and available tests results) and referred to secondary care do not have diagnosis confirmed following echocardiography and heart failure specialist review.^6^, ^10^, ^11^, ^12^

Heart failure continues to be misdiagnosed or underdiagnosed in primary care because GPs lack access to a uniform, low‐cost, and patient‐friendly ‘rule in’ test to objectively evaluate cardiac function.^7^, ^8^, ^13^ Diagnostic uncertainty results in diagnosis being delayed until symptoms are more obvious and more severe, requiring multiple consultations and hospital admissions, or people are treated incorrectly.^7^

The most recent definition of heart failure provided by the European Society of Cardiology suggests that heart failure results in reduced cardiac output at rest or during stress.^1^ Despite availability of non‐invasive, valid, and reliable methods for cardiac output assessment at rest and in response to stress,^14^, ^15^, ^16^, ^17^, ^18^ there were no recommendations from the guideline to incorporate measurement of cardiac output into the heart failure diagnostic pathway.^1^, ^2^ With a projected increase in prevalence of heart failure due to an ageing population, there will be an even greater pressure on echocardiography and cardiology services. Therefore, there is a huge clinical demand to equip GPs with a uniform, easy‐to‐use, low‐cost, and patient‐friendly test to objectively evaluate cardiac dysfunction and improve diagnostic accuracy of heart failure in primary care. In an attempt to address this clinical demand, we have recently developed a novel, easy‐to‐use, non‐invasive cardiac output response to stress (CORS) test in order to improve diagnosis and monitoring of heart failure in primary care. Prior further clinical (diagnostic and monitoring) effectiveness and cost‐effectiveness evaluation, it was considered prudent firstly to describe the CORS test and secondly to assess its reproducibility. Thus, the aim of the present study was to describe the CORS test for the first time and to investigate its reproducibility.

## Methods

### Study design, setting, and patients

The prospective observational study recruited 32 consecutive primary care patients (14 men and 18 women; mean age 63 ± 9 years) between February and November 2017. The study was conducted at the Clinical Research Facility of the Royal Victoria Infirmary, Newcastle upon Tyne, UK. The inclusion criteria were 50 years of age or more, no history of chronic, cardiovascular, pulmonary, or metabolic diseases, and willingness to visit the Clinical Research Facility. The exclusion criteria were inability to independently use the stairs and incapacity to provide written informed consent. The study protocol (number 15/NE/0190) was approved by the National Health Service, National Research Authority (North East – Tyne & Wear South Research Ethics Committee). All procedures performed in the study were in accordance with the Declaration of Helsinki. Participants gave written informed consent.

### Procedures

**Figure 1:**
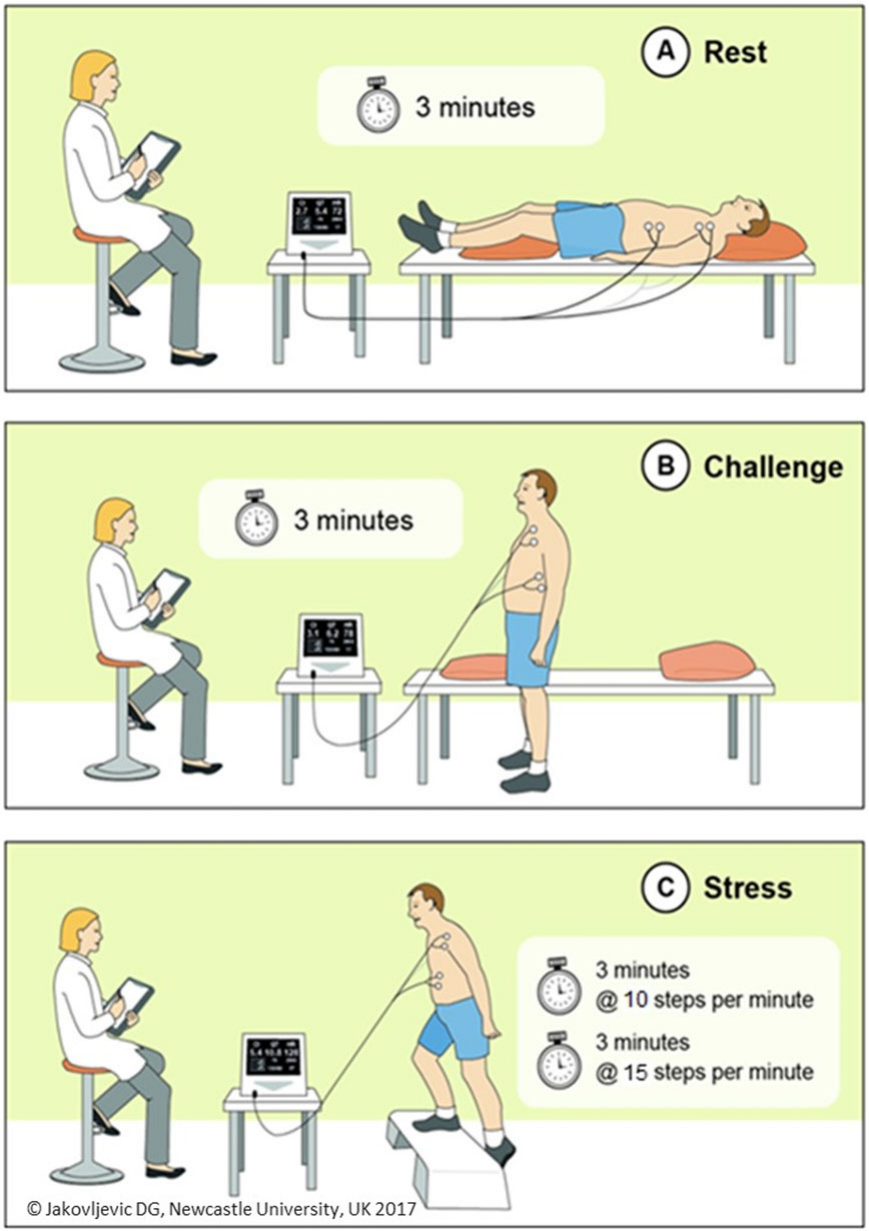
Cardiac output response to stress test. The cardiac output response to stress test consists of three phases: rest, challenge, and stress exercise. Each phase lasts for 3 min, and stress phase integrates additional 3 min to increase intensity and metabolic demand (from 10 to 15 steps per minute).

Participants attended the clinical physiology laboratory for one visit for approximately 2 h to complete the study (*Figure* *1*) and were instructed to abstain from vigorous exercise and alcohol consumption 24 h prior to the study visit. They were also asked to fast and not consume caffeine‐containing drinks for at least 2 h prior to the visit. Upon arrival at the laboratory, participants were asked to complete a standardized health screening questionnaire and physical examination to rule out any contraindications. This was followed by anthropometric measurements (body weight and height) and a 10 min rest period in supine position when arterial blood pressure and electrocardiography were performed.

### Study protocol and measurements

Cardiac function including cardiac output, cardiac index, heart rate, stroke volume, stroke volume index, blood pressure (systolic and diastolic), and mean arterial pressure were recorded at rest and during exercise using the non‐invasive cardiac output monitor, based on bioreactance technology (NICOM, Cheetah Medical, Inc., MA, USA), which we have previously evaluated.^14^, ^15^, ^16^, ^18^ This method uses four pairs of electrodes applied at the front side of the upper and lower thorax. Bioreactance is a novel method for continuous non‐invasive cardiac function monitoring and estimates cardiac output by analysing the frequency of relative phase shift of an electronic current delivered across the thorax. The CORS test (*Figure* *1*) includes measurements at rest and in response to stress (short exercise, i.e. step test). The CORS consists of four phases: 3 min rest (supine), 3 min challenge (standing), and two 3 min step‐exercise test stages (i.e. Stage 1 step exercise, 10 steps/minute followed by Stage 2 step exercise, 15 steps/minute) using a 15 cm height bench. The online metronome (TempoPerfect Metronome Software, NCH Software, CO, USA) was used to guide participants step frequency during the step‐exercise test, i.e. 10 and 15 steps per minute. To evaluate reproducibility of the CORS test, it was repeated twice (referred to as Test 1 and Test 2) with at least 1 h sitting rest between the two tests. Longer rest time was implemented if participants' resting metabolic and haemodynamic measurements had not returned to their baseline values obtained before Test 1. Simultaneously with cardiac output and automated blood pressure measurements, gas exchange metabolic data were also collected (i.e. oxygen consumption and carbon dioxide production) at rest and during exercise using the metabolic analyser (Cortex, Leipzig, Germany). Measurement of gas exchange data was performed in order to evaluate the metabolic demand of the CORS test.

### Sample size

It was estimated that recruitment of 32 study participants will be sufficient to allow evaluation of the CORS test reproducibility. The CORS test includes three phases (rest, challenge, and exercise, with exercise including two sub‐phases). It was calculated that the total number of assessment points will be 128, which is obtained by multiplying 32 patients and 4 data collection points. It was considered that 128 repeated measures of cardiac function will be sufficient to allow detailed evaluation of the reproducibility of the CORS test using statistical methods described in the next section.

### Statistical analysis

Data were analysed using SPSS, version 24 (SPSS, Inc., Chicago, IL, USA). The level of significance was set at *P* < 0.05. Data are expressed as mean (SD). Reproducibility of haemodynamic and metabolic variables was calculated using coefficient of variation (CV), while linear relationships between repeated measures were assessed using Pearson's correlation coefficient (*r*). The CV was calculated as a percentage of within‐person standard deviation divided by within‐person average. A CV of ≤6% was considered as good reproducibility, while CV of 6–10% and >10% was considered acceptable and poor reproducibility, respectively.^19^ Additionally, Bland–Altman plots were constructed to evaluate the upper and lower limits of agreements (±2 SD of mean difference) of cardiac output measured at rest and at different intensities of exercise.^20^

## Results

Participant demographic and clinical characteristics are detailed in *Table* 1. Out of 32, seven participants (22%) were prescribed with antihypertensive medication; two were prescribed with antidepressants and lipid‐lowering medication. Physical characteristics of the participants were as follows: age 63 (range: 50–89) years, weight 71 (45–102) kg, height 165 (150–180) cm, and body mass index 26 (21–32) kg/m^2^. All participants completed all phases of the CORS test without any contraindication highlighting acceptability for completing this test in this study group. Excellent quality of the signal for cardiac output measurement was obtained in 30 (97%) participants, giving a total of 126/128 data points when data from all phases were combined. The NICOM signal was lost for two participants during Stage 1 step exercise. Complete metabolic data were obtained in 28 (88%) participants, (112/128 data points; due to fault in the sample line causing missing metabolic data).

**Table 1:** Participant demographics and clinical characteristics

Number of participants (*N*)	32
Age (years)	63 ± 9
Male, *n* (%)	14 (44)
Height (cm)	165 ± 8
Weight (kg)	71 ± 13
Body mass index (kg/m^2^)	26 ± 4
Medications *N* (%**)**	
Antidepressant	2 (6)
β_2_ receptor agonist	1 (3)
Bile acid sequestrant	1 (3)
Angiotensin‐converting enzyme inhibitor	2 (6)
Calcium channel blocker	3 (9)
Loop diuretic	1 (3)
Acetylsalicylic acid	1 (3)
Anticoagulant	1 (3)
Benign prostatic hyperplasia	1 (3)
Statins	1 (3)
Angiotensin‐converting enzyme inhibitor	1 (3)
Anticholinergic	1 (3)

**Figure 2:**
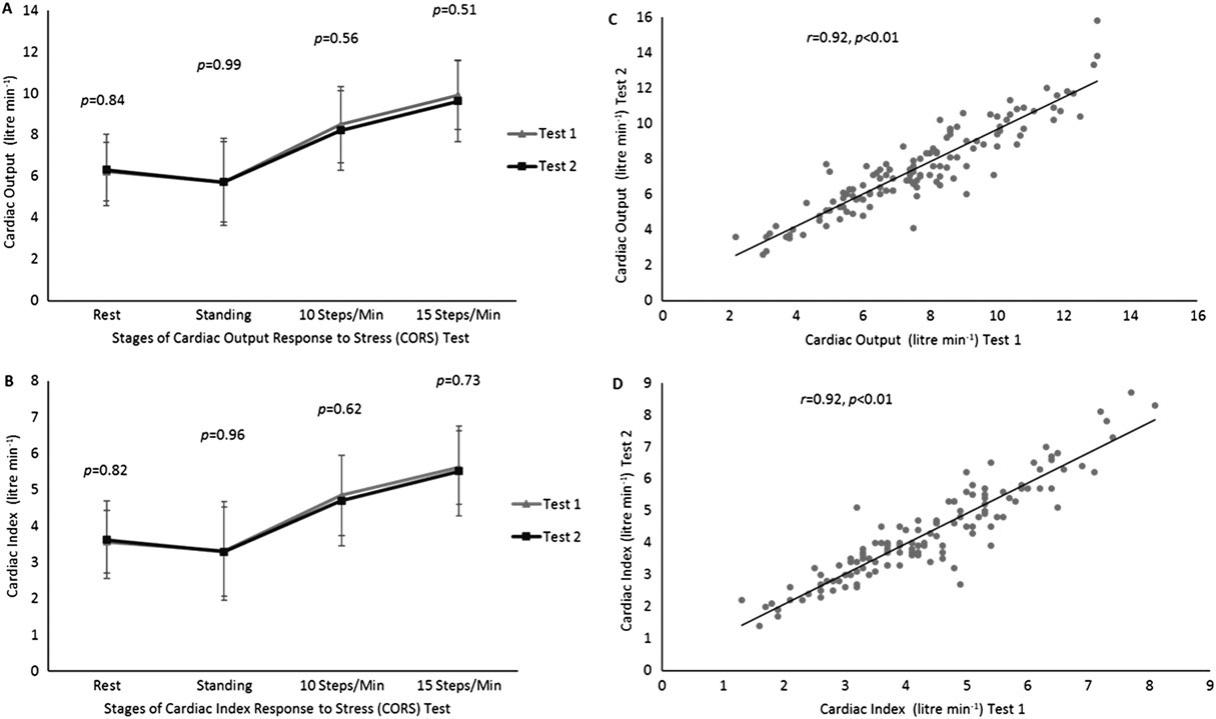
Mean cardiac output and cardiac index values and relationship between repeated cardiac output and cardiac index values: (A) mean cardiac output for each stage of the cardiac output response to stress (CORS) test for Test 1 and Test 2; (B) mean cardiac index for each stage of the CORS test for Test 1 and Test 2; (C) relationship between cardiac output at Test 1 and Test 2 when all data are grouped together (CORS test); (D) relationship between cardiac index at Test 1 and Test 2 when all data are grouped together (CORS test).

There was no significant difference in resting, standing, and step‐exercise haemodynamic and metabolic data between Test 1 and Test 2 (*Tables* 2 and 3, respectively). At all phases of the CORS test, there were no significant differences in either cardiac output or cardiac index values between Test 1 and Test 2 (*Figure* *2* *A* and *Figure* *2* *B*). There was a strong relationship between Test 1 and Test 2 cardiac outputs when all phase data (rest, standing, and Stage 1 and Stage 2 step exercises) were grouped together (*r* = 0.92, *P* = 0.01) (*Figure* *2* *C*). Similarly, when all data were grouped, a strong relationship was found between Test 1 and Test 2 for cardiac index when all data were grouped together (*r* = 0.92, *P* = 0.01) (*Figure* *2* *D*).

**Table 2:** Reproducibility of haemodynamic measures

Variables	Test 1	Test 2	P	*r*	CV (%)
Rest					
QT (L/min)	6.2 (1.4)	6.3 (1.7)	0.84	0.80	8.1
CI (L/min/m^2^)	3.6 (0.9)	3.6 (1.1)	0.82	0.78	9.0
HR (beats/min)	62 (7)	59 (8)	0.18	0.91	3.9
SV (mL/beat)	102 (24)	108 (32)	0.36	0.82	9.3
SVI (mL/beat/m^2^)	58 (14)	62 (19)	0.38	0.80	9.8
SBP (mmHg)	126 (12)	129 (15)	0.39	0.74	4.5
DBP (mmHg)	81 (11)	80 (11)	0.73	0.85	4.1
MAP (mmHg)	95 (12)	96 (11)	0.62	0.76	4.2
Standing					
QT (L/min)	5.7 (2.1)	5.7 (1.9)	0.99	0.94	6.4
CI (L/min/m^2^)	3.3 (1.4)	3.3 (1.2)	0.96	0.95	6.5
HR (beats/min)	71 (8)	70 (9)	0.64	0.89	3.0
SV (mL/beat)	82 (32)	83 (29)	0.93	0.94	6.6
SVI (mL/beat/m^2^)	47 (19)	47 (18)	0.96	0.94	6.8
SBP (mmHg)	132 (17)	130 (16)	0.60	0.80	4.5
DBP (mmHg)	87 (11)	87 (10)	0.81	0.91	2.8
MAP (mmHg)	102 (11)	101 (11)	0.87	0.96	1.6
Stage 1 exercise (10 steps/minute)					
QT (L/min)	8.5 (1.8)	8.2 (1.9)	0.56	0.85	6.7
CI (L/min/m^2^)	4.8 (1.1)	4.7 (1.2)	0.62	0.90	6.5
HR (beats/min)	83 (11)	80 (11)	0.33	0.95	3.1
SV (mL/beat)	104 (26)	104 (27)	0.99	0.89	6.1
SVI (mL/beat/m^2^)	59 (16)	59 (17)	0.96	0.90	6.3
SBP (mmHg)	154 (23)	143 (18)	0.05	0.88	5.6
DBP (mmHg)	84 (12)	82 (10)	0.50	0.84	4.2
MAP (mmHg)	107 (13)	102 (11)	0.12	0.88	4.3
Stage 2 exercise (15 steps/minute)					
QT (L/min)	9.9 (1.7)	9.6 (2.0)	0.51	0.79	7.1
CI (L/min/m^2^)	5.6 (1.0)	5.5 (1.2)	0.73	0.87	6.5
HR (beats/min)	90 (12)	88 (13)	0.53	0.96	2.4
SV (mL/beat)	109 (29)	111 (26)	0.76	0.79	8.3
SVI (mL/beat/m^2^)	64 (14)	64 (16)	0.95	0.88	6.8
SBP (mmHg)	158 (24)	148 (21)	0.12	0.76	5.5
DBP (mmHg)	82 (12)	81 (11)	0.65	0.86	4.2
MAP (mmHg)	107 (12)	103 (12)	0.19	0.81	3.9

CI, cardiac index; CV, coefficient of variation; DBP, diastolic blood pressure; HR, heart rate; MAP, mean arterial pressure; QT, cardiac output; *r*, correlation coefficient; SBP, systolic blood pressure; SV, stroke volume; SVI, stroke volume index.

Data are expressed as mean (SD).

**Table 3:** Reproducibility of metabolic measurements

Variables	Test 1	Test 2	*P* value	*r*	CV (%)
Rest					
VO_2_ (L/min)	0.3 (0.1)	0.3 (0.1)	0.87	0.62	13.5
VO_2_ (mL/kg/min)	3.5 (1.0)	3.4 (1.0)	0.90	0.53	12.5
VCO_2_ (L/min)	0.2 (0.1)	0.2 (0.1)	0.87	0.77	11.0
RER	0.8 (0.1)	0.8 (0.1)	0.99	0.65	4.5
VE (L/min)	6.7 (2.1)	6.7 (2.4)	0.90	0.70	13.5
VE/VCO_2_	28.1 (2.9)	27.2 (3.5)	0.27	0.57	6.7
BF	11.8 (2.9)	11.4 (3.3)	0.61	0.43	14.4
Standing					
VO_2_ (L/min)	0.3 (0.1)	0.3 (0.1)	0.74	0.63	14.4
VO_2_ (mL/kg/min)	3.7 (1.0)	3.7 (1.1)	0.97	0.54	15.0
VCO_2_ (L/min)	0.2 (0.1)	0.2 (0.1)	0.81	0.78	14.0
RER	0.8 (0.1)	0.8 (0.1)	0.38	0.86	3.2
VE (L/min)	8.3 (3.0)	8.0 (3.2)	0.80	0.75	13.6
VE/VCO_2_	31.8 (5.0)	31.2 (4.5)	0.68	0.86	4.3
BF	13.8 (3.4)	13.4 (3.2)	0.68	0.79	7.6
Stage 1 exercise (10 steps/minute)					
VO_2_ (L/min)	0.7 (0.2)	0.7 (0.2)	0.81	0.46	14.8
VO_2_ (mL/kg/min)	10.4 (2.4)	9.9 (2.1)	0.41	0.51	11.2
VCO_2_ (L/min)	0.5 (0.2)	0.5 (0.2)	1.00	0.61	14.7
RER	0.7 (0.1)	0.7(0.1)	0.55	0.18	5.8
VE (L/min)	16.1 (4.7)	15.2 (5.2)	0.47	0.78	11.5
VE/VCO_2_	28.2 (2.8)	27.6 (2.6)	0.37	0.56	4.4
BF	18.3 (2.7)	17.3 (2.6)	0.16	0.49	9.1
Stage 2 exercise (15 steps/minute)					
VO_2_ (L/min)	0.9 (0.2)	0.8 (0.2)	0.70	0.76	8.5
VO_2_ (mL/kg/min)	12.5 (2.2)	11.8 (2.6)	0.25	0.84	6.9
VCO_2_ (L/min)	0.7 (0.2)	0.6 (0.2)	0.47	0.79	10.7
RER	0.8 (0.1)	0.8 (0.0)	0.47	0.88	2.2
VE (L/min)	20.2 (7.4)	18.7 (6.1)	0.42	0.91	8.7
VE/VCO_2_	27.5 (2.7)	26.1 (2.3)	0.11	0.82	3.1
BF	18.9 (3.4)	17.3 (4.7)	0.17	0.77	6.7

BF, breathing frequency; CV, coefficient of variation; *r*, correlation coefficient; RER, respiratory exchange ratio; VE, minute ventilation; VE/VCO_2_, minute ventilation and carbon dioxide production; VCO_2_, carbon dioxide release; VO_2_, oxygen consumption.

Data are expressed as mean (SD).

When all data from all stages of the CORS test were combined, the CVs for cardiac output, cardiac index measurements, and oxygen consumption (mL/kg/min) were 7.1%, 7.0%, and 10.0%, respectively (*Tables* 2 and 3).

**Figure 3:**
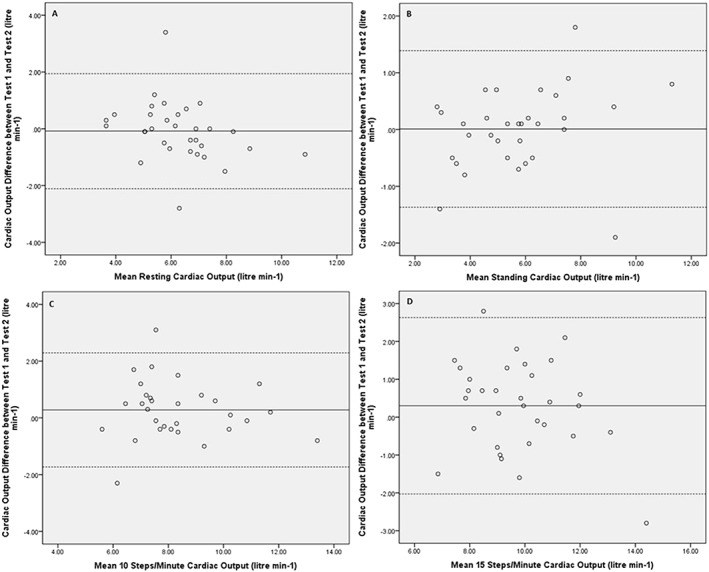
Bland–Altman plots to demonstrate limits of agreement for cardiac output between Test 1 and Test 2 at each of four stages of the cardiac output response to stress test, i.e. (A) at rest, (B) standing, and the 3 min stages of step exercise, (C) 10 steps per minute and (D) 15 steps per minute. The solid line represents the mean difference, and the dashed lines represent lower and upper limits of agreement between Test 1 and Test 2.

Bland–Altman analyses were performed to demonstrate the agreement between Test 1 and Test 2 cardiac outputs for each stage of the CORS test (*Figure* *3*). When all data were combined, the mean difference and lower and upper limits of agreement between Test 1 and Test 2 cardiac outputs were 0.1 (−1.9 to 2.1 L/min; *Figure* *5* *A*).

**Figure 4:**
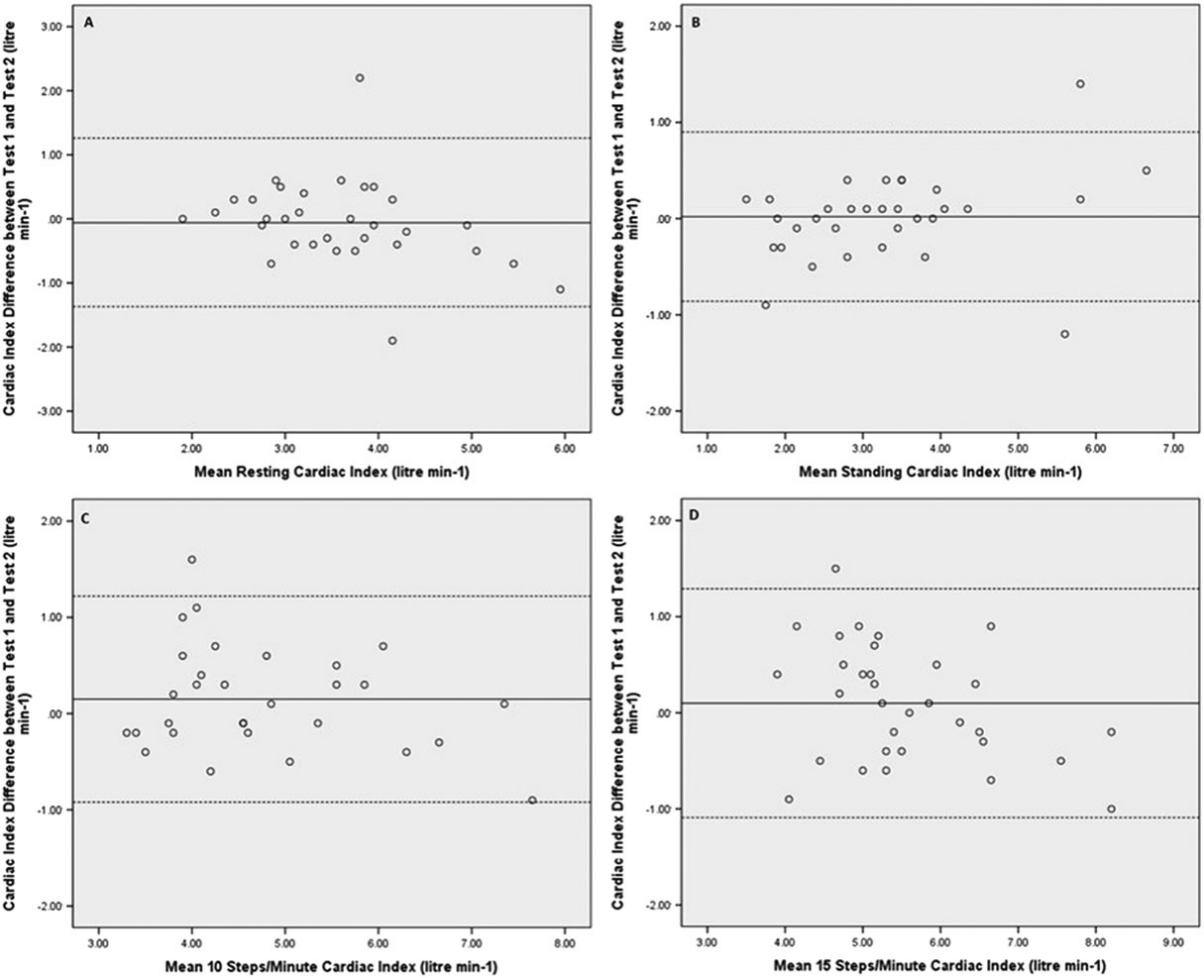
Bland–Altman plots to demonstrate limits of agreement for cardiac index between Test 1 and Test 2 at each of the four stages of the cardiac output response to stress test, i.e. (A) at rest, (B) standing, and the 3 min stages of step exercise, (C) 10 steps per minute and (D) 15 steps per minute. The solid line represents the mean difference, and the dashed lines represent lower and upper limits of agreement between Test 1 and Test 2.

**Figure 5:**
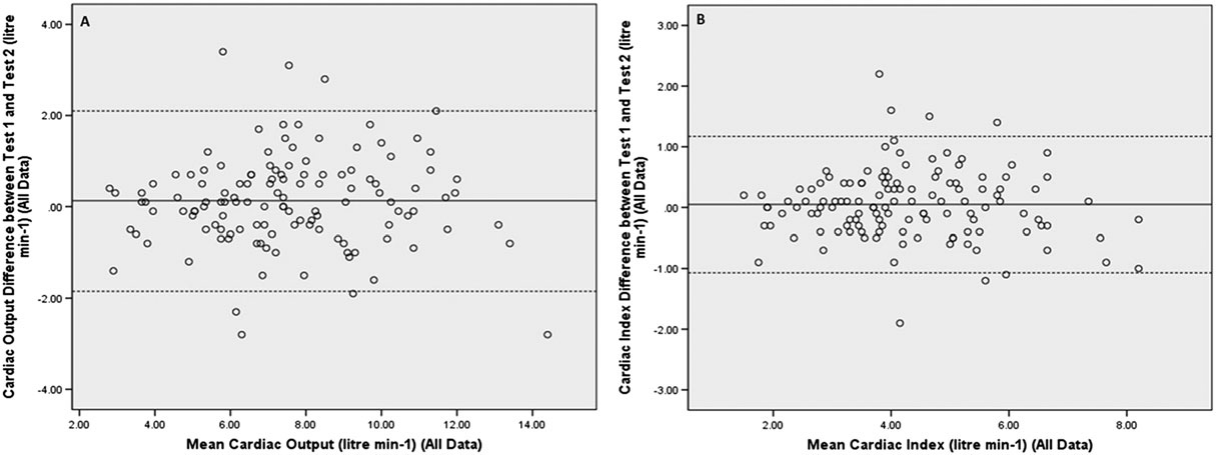
Bland–Altman plots to demonstrate limits of agreement between Test 1 and Test 2 for all stages of the cardiac output response to stress test: (A) combined data for cardiac output and (B) combined data for cardiac index. The solid line represents the mean difference, and the dashed lines represent lower and upper limits of agreement between Test 1 and Test 2.

Cardiac index was also not significantly different between the two tests (as shown in *Table* 2 and *Figure* *2* *D*) with Bland–Altman analyses suggesting acceptable limits of agreement (*Figure* *4*). When all data were combined, the mean cardiac index difference between the two tests was −0.1 L/min/m^2^ with lower and upper limits of agreeing −1.1 and 1.2 L/min/m^2^, respectively (*Figure* *5* *B*).

## Discussion

### Statement of principal findings

The purpose of this study was to firstly describe the CORS test and secondly to investigate its reproducibility. The major findings of the current study suggest that the CORS test demonstrated acceptable level of reproducibility. A strong positive relationship was found between repeated key measures of the CORS test, i.e. cardiac output and cardiac index. The calculated limits of agreement were narrow and acceptable for each stage of the CORS test for both cardiac output and cardiac index.

### Strengths and weaknesses of the study

For the first time, the present study describes a novel CORS test developed with the potential to improve diagnosis and monitoring of heart failure in primary care. Haemodynamic measurements, i.e. cardiac output and cardiac index, are coupled with metabolic gas exchange measurements allowing accurate determination of the CORS' metabolic demand.

The following limitations should be considered. It was not possible to use a gold standard method for measuring cardiac output (i.e. thermodilution or direct Fick method) due to their invasive approach and associated risks. Previous studies demonstrated a positive relationship between cardiac output estimates obtained from bioreactance and thermodilution methods in patients undergoing surgical interventions.^21^, ^22^ Secondly, although the overall sample size was small to moderate, it should be noted that multiple measurements and cardiac output values were obtained at different body positions as well as two different exercise intensities, which provide sufficient data points (>120) for detailed reproducibility analysis to be performed. Lastly, the sample population did not include patients with suspected or confirmed diagnosis of heart failure. However, our ongoing research programme aims to confirm clinical utility of the CORS test to improve diagnosis and monitoring of heart failure in primary care.

### Strengths and weaknesses in relation to other studies, discussing important differences in results

Overall, a CV of ~7% was found for both cardiac output and cardiac index, respectively, suggesting acceptable reproducibility of the CORS test.^23^ Reproducibility of haemodynamic measures at rest and their response to stress in heart failure using a non‐invasive gas rebreathing technology has been previously reported.^19^ In post‐cardiac surgery patients, the bioreactance method was validated against thermodilution with a strong relationship reported between the two methods.^24^ Myers *et al*. also found strong relationships between peak cardiac index and peak oxygen consumption when measured by direct Fick and bioreactance method in patients with heart failure.^25^ At rest and in response to stress exercise, the bioreactance method has previously demonstrated good test–retest reliability for estimating cardiac output.^15^ This further supports the use of the bioreactance method and its integration into the CORS test to estimate cardiac output at rest and in response to stress exercise as used in the present study with the overall aim to improve diagnosis and monitoring of heart failure in primary care.

Cardiac index declines with age but does not differ between male and female healthy individuals.^26^ The same study suggests that 50% of patients with heart failure reduced ejection fraction demonstrate reduced cardiac index at rest compared with healthy controls.^26^ However, it appears that both heart failure patients with reduced as well as preserved ejection fraction demonstrate diminished cardiac output (cardiac index) in response to exercise‐induced stress.^27^, ^28^, ^29^

Data from the present study indicate reproducibility of the CORS test as the relationship between repeated cardiac output measures were strong. Similar strength of the relationship has been previously reported for bioreactance cardiac outputs at rest and in response to maximal graded cardiopulmonary exercise testing procedure.^15^ Bland–Altman analyses for cardiac output and cardiac index demonstrated low mean differences between Test 1 and Test 2 and acceptable limits of agreement for each stage of the CORS test when all data were grouped together. A recent comparison study between cardiac output estimated by bioreactance and gas rebreathing during exercise recently demonstrated acceptable limits of agreement between the two methods.^18^

These findings suggest that the bioreactance method, as used in the present study and integrated in the CORS test, is a reliable method in estimating cardiac output at rest and in response to stress exercise.

### Meaning of the study: explanations and implications for clinicians and policymakers

According to the most recent European Society of Cardiology guideline, heart failure is presented with reduced cardiac output at rest and/or in response to stress.^1^ Accordingly, measurement of cardiac output not only at rest but particularly during short stress, such as exercise, can help detect heart failure. An accurate measure of cardiac output is essential for differentiating diagnosis in many clinical presentations and specialities including anaesthesiology, emergency care, and cardiology.^16^, ^30^ Enhancing early diagnosis of heart failure in primary care using the CORS as a ‘rule in’ test (to be performed in natriuretic peptide positively tested patients) may (i) reduce the number of inaccurate and expensive referrals to secondary care, which worry patients, and (ii) improve patient quality and length of life and reduce the number of hospital admissions as it will allow early administration of evidenced‐based proved to improve symptoms and outcomes.^1^

Echocardiography (performed in secondary care) with cardiology heart failure specialist review remains the reference method for the diagnosis of heart failure.^1^, ^2^ However, the routine use of echocardiography in primary care setting is limited due to time, required expertise, and facilities.^31^ Serum natriuretic peptide tests continue to be used as a ‘rule out’ test to exclude heart failure in clinical practice, but reduced specificity and inconsistencies in optimal thresholds lead to misdiagnosis and inaccurate and expensive referrals from primary to secondary care.^1^, ^2^, ^9^, ^32^

### Unanswered questions and future research

Findings of the present study demonstrate acceptable reproducibility of the CORS test to evaluate cardiac output at rest and in response to exercise stress. Our ongoing research will determine clinical (diagnostic accuracy) effectiveness and cost‐effectiveness of the CORS to be used as a ‘rule in’ test in primary care to improve diagnosis and referral accuracy of patients with suspected heart failure. Future implementation of the CORS in primary care will lead to improved length and quality of life for patients and reduced number of inaccurate and expensive referrals, which are worrying for patients. The current National Institute for Clinical Excellence guideline for diagnosis and management of heart failure in primary and secondary care and clinical practice suggests that heart failure as a final diagnosis is only confirmatory in only ~1/3 of patients suspected of having disease.^2^ Previous evidence suggest that general practice records indicate that only 34% of patients with an existing clinical label of heart failure had diagnosis confirmed at echocardiography and specialist review^4^ and that average waiting time for this investigation was 67 days.^33^ However, early diagnosis of heart failure improves short‐term and long‐term outcomes while reducing the risk of hospital admissions by 23%.^2^ Considering poor prognosis is consequential of a delayed diagnosis of heart failure, and the lack of additional testing tools in primary care to better identify cardiac dysfunction, it is reasonable to suggest that the CORS test may have an important role within primary care to improve diagnosis of heart failure and help GPs monitor patients and their response to treatment.

## Conclusions

The present study for the first time describes a novel, easy‐to‐use, patient‐friendly, non‐invasive CORS test. Major findings suggest acceptable reproducibility of the CORS test, which is developed to potentially improve diagnosis and monitoring of heart failure in primary care. Our ongoing research investigates clinical effectiveness and cost‐effectiveness of the CORS test to be used by primary care physicians as a ‘rule in’ test to improve diagnosis and monitoring of heart failure in primary care.

## Conflict of interest

The authors have no relationships or activities that could appear to have influenced the submitted work.

## Author contributions

D.G.J. and G.A.M. conceived the study concept and design and supervised the study; S.J.C., N.C.O., R.J.S., G.A.M., K.B., J.S., and D.G.J. acquired the data; S.J.C. and D.G.J. performed the statistical analysis and interpreted the data and provided administrative, technical, or material support; S.J.C., N.C.O., and D.G.J. drafted the manuscript; K.B., J.A., A.R., P.M.S., M.S., S.T., A.F., J.M., F.D.R.H., and G.A.M. revised the manuscript critically for important intellectual content. All authors critically reviewed the manuscript and approved the final version. D.G.J. acts as the guarantor and takes responsibility for the content of the manuscript, including the data and analysis.

## Funding

This study was funded by the UK Medical Research Council Confidence in Concept Scheme grant to D.G.J. (grant no. BH161161). D.G.J. is supported by the UK Research Councils' Newcastle Centre for Ageing and Vitality (grant no. L016354). The views expressed are those of the authors and not necessarily those of the Medical Research Council. The funders of the study had no role in study design or in data collection, analysis, or interpretation.

## Ethical approval

The study protocol (number 15/NE/0190) was approved by the National Health Service, National Research Authority (North East – Tyne & Wear South Research Ethics Committee).

## Data sharing

The relevant anonymized patient level data are available on reasonable request from the authors.
