# Corrigendum

**DOI:** 10.1002/ehf2.12441

**Published:** 2019-04-25

In the paper by Charman *et al.,* 1 R.F.D. Hobbs's affiliations should have included:

NIHR Oxford Biomedical Research Centre, Oxford University Hospitals NHS Foundation Trust.

In addition, the Acknowledgements should have appeared as follows.
